# Grid Frequency Measurement through a PLHR Analysis Obtained from an ELF Magnetometer

**DOI:** 10.3390/s22082954

**Published:** 2022-04-12

**Authors:** Francisco Portillo, Alfredo Alcayde, Rosa M. García, Nuria Novas, José Antonio Gázquez, Manuel Férnadez-Ros

**Affiliations:** Department of Engineering, University of Almeria, ceiA3, 04120 Almeria, Spain; portillo@ual.es (F.P.); aalcayde@ual.es (A.A.); rgarciasalvador@ual.es (R.M.G.); nnovas@ual.es (N.N.); mfernandez@ual.es (M.F.-R.)

**Keywords:** PLHR, frequency measurement, zero-crossing, FFT

## Abstract

The stability of the power grid’s frequency is crucial for industrial, commercial, and domestic applications. The standard frequency in Europe’s grid is 50 Hz and it must be as stable as possible; therefore, reliable measurement is essential to ensure that the frequency is within the limits defined in the standard EN 50160:2010. In this article, a method has been introduced for the measurement of the grid frequency through a power line harmonics radiation analysis. An extremely low-frequency magnetometer was developed with the specific purpose of monitoring, in real time, the electromagnetic field produced by electrical installations in the range from 0 to 2.2 kHz. Zero-crossing and Fast Fourier transform algorithms were applied to the output signal to calculate the grid frequency as a non-invasive method. As a final step, data for a complete month (May 2021) were compared with a commercial power quality analyzer connected to the main line to validate the results. The zero-crossing algorithm gave the best result on 3 May 2021, with a coefficient of determination (R^2^) of 0.9801. Therefore, the indirect measurement of the grid frequency obtained through this analysis satisfactorily fits the grid frequency.

## 1. Introduction

Most of the electric power systems of the world operate with a fundamental frequency of 50 or 60 Hz, depending upon the country [1]. This system carries electricity from the generation centers to industrial, commercial, and domestic users, but they also generate electromagnetic fields (EMF) at their fundamental frequency and at their harmonics, commonly called power line harmonics radiations (PLHR). If the radiation refers only to the fundamental component, it is usually called power line emission (PLE) [2].

**Figure 1:**
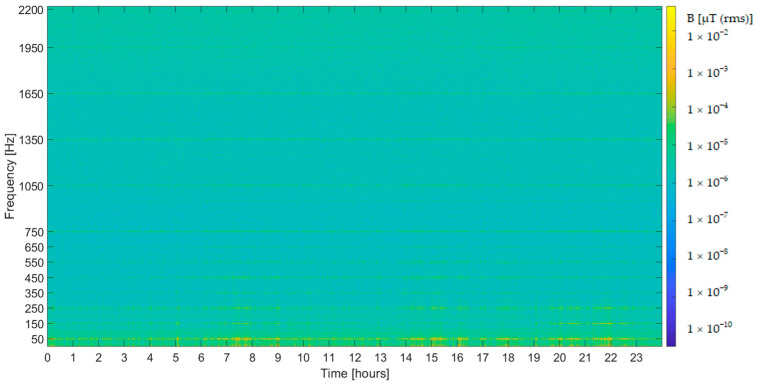
Frequency–time spectrogram captured by an ELF magnetometer on 3 May 2021.

PLHR are commonly illustrated in a form of frequency-time spectrograms with color-coded power spectral density of EMF fluctuations. They take an arrangement of a set of discrete horizontal lines where the frequency separation depends on the fundamental frequency of the electrical system in the generation area [3]. Figure 1 shows a spectrogram of the PLHR captured by an extremely low frequency (ELF) magnetometer, where there are visible lines at the fundamental frequency of 50 Hz and its harmonics.

Weak but coherent signals can be amplified and can trigger emissions when they reach the wave–particle interaction region near the equator (about 30 dB above the input signal strength) [4]. The physical mechanism responsible for the triggered very-low-frequency emissions is the electron cyclotron resonance between these waves and the keV radiation belt electrons [5]. In 1975, Helliwell et al. [6] performed some experiments at the Siple Station, Antarctica, and at its magnetic conjugate point, in the industrialized region of Roberval, Canada. They identified the PLHR lines for the first time and postulated that their origin was in the radiation of the terrestrial power lines.

**Figure 2:**
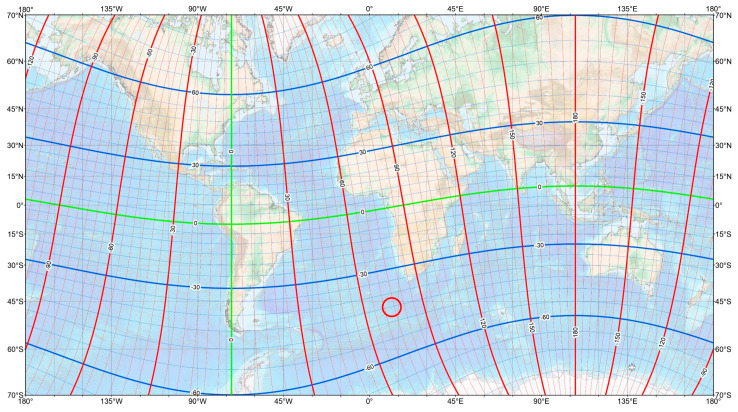
Geomagnetic coordinates map [9] with the conjugate point of Spain in a red circle.

Considering the underlying mechanism of the PLHR amplification in the ionosphere, the only possible source of the PLHR in Spain is found in its electrical system, since the magnetic conjugate of the Iberian Peninsula is located in the southern Atlantic Ocean (red circle in Figure 2), far from any power system. In the lower atmosphere, a fact that must be emphasized about the propagation of the PLHR is the low attenuation of the ELF band radiation. According to Burke and Jones, the attenuation at 40 Hz frequency is about 0.64 dB/1000 km, although there is some dependence on the latitude [7]. This fact allows these waves to propagate with very little attenuation. Other authors [8] have carried out a similar work using simulations and analytical methods that corroborate this low attenuation. However, the lack of data above 50 Hz limits the ability of these methods to obtain reliable results beyond the fundamental frequency of the power grid.

The contributions in the field of PLHR are doubled every decade [10], and this research continues in several areas, such as engineering, astrophysics, medicine, and even biology [11]. PLHR are a source of man-made electromagnetic pollution [12], and they have been accepted as an electromagnetic compatibility (EMC) problem [2], especially in industrial areas. The universal development of the electricity network has led to a growth in electromagnetic pollution. Only in Spain, the electricity distribution network reached 44,471 km in 2020 [13], which is generating the consequent concern in the population about human exposure to the latent danger inherent in the ELF radiation. As a recognized man-made pollutant, the environmental effect caused by PLHR is becoming an increasing cause of concern to the public and experts alike [14], which raises the need for large-scale PLHR research [15]. This has led many scientists and researchers to investigate the potential effects and risks to human health [16], with most of the publications being concentrated in the last decades [17].

It was in 1970 when a study tried to clarify the effects of the ELF radiation in animals, specifically in laboratory rats [18], although no significant differences were found between rats exposed to radiation and the control group. The major concern in this field began as a result of an epidemiological study carried out in 1979 [19]. The authors found a relationship between the risk of childhood leukemia and an indirect measure of the degree of exposure to ELF radiation from power transmission lines. Subsequent studies found no evidence to justify the high level of media speculation about possible harmful effects [20]. One of the most cited studies by the scientific community on the influence of low-frequency EMF on human health is a review article from 1998 [21]. The researchers, after reviewing the evidence accumulated from epidemiological studies up to that date, concluded that a clear correlation between ELF radiation exposure and cancer development could not be provided.

The limits of exposure to ELF radiation proposed by the standards are becoming less and less restrictive since no study has been able to demonstrate the relationship between the effects of exposure to these fields and possible adverse effects on human health. One of the objectives promoted by the World Health Organization is that exposure to EMF, whether for workers or the public, should be monitored to ensure that appropriate exposure limits are not exceeded [22]. Currently, many research articles are focused on EMF measurements campaigns in various environments, such as near power lines and substations [23], urban, homes and work environments [24], industries [25], etc.

However, PLHR could not only affect human health. It is commonly believed that the effects are limited only to the Earth’s surface and atmosphere, but they could also reach large parts of the near-Earth space environment [26], polluting the ionosphere and magnetosphere. In this way, to enhance the knowledge and expertise of PLHR, the R&D project UAL18-TIC-A025-A called “Monitored Electromagnetic Field Generated by Electrical Grids” was created within the framework of the Andalusia Operational Program 2014–2020 of the European Regional Development Fund (ERDF).

The goal of monitoring the EMF produced by equipment and electrical installations in real time is due to the fact that PLHR affect the quality of the energy, damage the regular operation of the electrical network, and disturb the communications system [27,28]. Despite this, the users of the electrical grid are entitled to services of high quality and it is essential for a stable and reliable supply of electrical power for both society and the economy [29]. To ensure this, some criteria are established in a series of specific regulations and guides. The most often used standard related to power quality is EN 50160:2010 “Voltage characteristics of electricity supplied by public distribution networks” [30] with further amendments EN 50160:2010/A1:2015 [31], EN 50160:2010/A2:2019 [32], and EN 50160:2010/A3:2019 [33].

This European Standard defines, describes, and specifies the main characteristics of the voltage at a network user’s supply terminals in public high, medium, and low voltage AC electricity networks under normal operating conditions. It describes the limits or values within which the voltage characteristics can be expected to be maintained at any point of supply in the general distribution network, but it does not describe the average situation normally experienced by an individual network user. The goal of this standard is to define, describe, and specify the characteristics of the voltage, such as frequency, amplitude, waveform, and symmetry of the line voltages.

One of the most important parameters in the quality of the electricity network is the frequency [34] which must be kept as stable as possible. The standard value in Europe’s electricity grid is 50 Hz, and to ensure that the frequency always remains at a stable level, the balance between consumption and production of electrical power must always be right. A mismatch of consumption and production induces frequency deviations from the nominal frequency [35]. Nevertheless, these deviations are low if the different networks are interconnected to achieve a very large production capacity compared to the variations that may occur [30]. Regarding this matter, a recent problem is the additional fluctuations in frequency that the massive implementation of wind or photovoltaic generation introduces [36], reducing the overall inertia available in the grid [37]. This is due to the absence of inertial response and auxiliary frequency support [38] in both wind and solar, so the all-important damping effect is missing.

According to the standard for high, medium, and low voltage networks with synchronous connection to an interconnected system and under normal conditions of operation, the mean frequency measured in periods of 10 s should be:

- 50 Hz ±1% (49.5 to 50.5 Hz) for 99.5% of the year;
- 50 Hz −6%/+4% (47 to 52 Hz) for 100% of the time.

Amendment A2 [32] added that under exceptional circumstances, temporarily wider frequency tolerances may be applied with the purpose of securing an electricity supply.

The entire electrical power system has become more and more complex in recent decades due to the connection of non-linear loads, the use of distributed generation, and the presence of unexpected faults [34]. Power networks are often close to their stability limits because they are operated under heavily loaded conditions [39]. Therefore, observation becomes mandatory as electrical demand continues to increase [40].

Power system stability is defined as the property of a power system to remain in an operating equilibrium state under normal operating conditions and to return to its normal or stable conditions after being disturbed. In this regard, frequency stability is considered a critical issue for power system operation [41]. A permanent deviation of the frequency from its nominal value may affect power system operation, reliability, security, and efficiency by damaging equipment, degrading load performance, overloading transmission lines, and triggering the electrical protection devices [42]. Accordingly, frequency measurement is a matter of crucial importance in the field of electrical engineering.

The measurement methods applied in this standard are described in standard IEC 61000-4-30:2015 “Electromagnetic compatibility (EMC)-Part 4-30: Testing and measurement techniques-Power quality measurement methods” [43] and the erratum IEC 61000-4-30:2015/COR1:2016 [44]. This standard defines the measurement methods and the interpretation of the results for power quality parameters in AC power supply systems with a declared fundamental frequency of 50 Hz or 60 Hz. The measurement methods are described for each type of parameter in terms that give reliable and repeatable results, regardless of the method’s implementation.

Taking all the above into account, a procedure for measuring the grid frequency through a PLHR analysis has been investigated. The dataset used in this article was part of a broader collection from the R&D project UAL18-TIC-A025-A mentioned above that originally was created with the aim of monitoring, in real time, the EMF produced by equipment and electrical installations ranging from 0 to 2.2 kHz. In that way, the grid frequency was measured in a non-invasive way.

## 2. Materials and Methods

We begin by describing the materials and methods used in this study, followed by the algorithms. For data processing, MatLab^®^ R2021.a (MA, USA) was used in this work. The selected data correspond to a complete month of 2021, specifically from 1 May to 31.

### 2.1. Data

**Figure 3:**
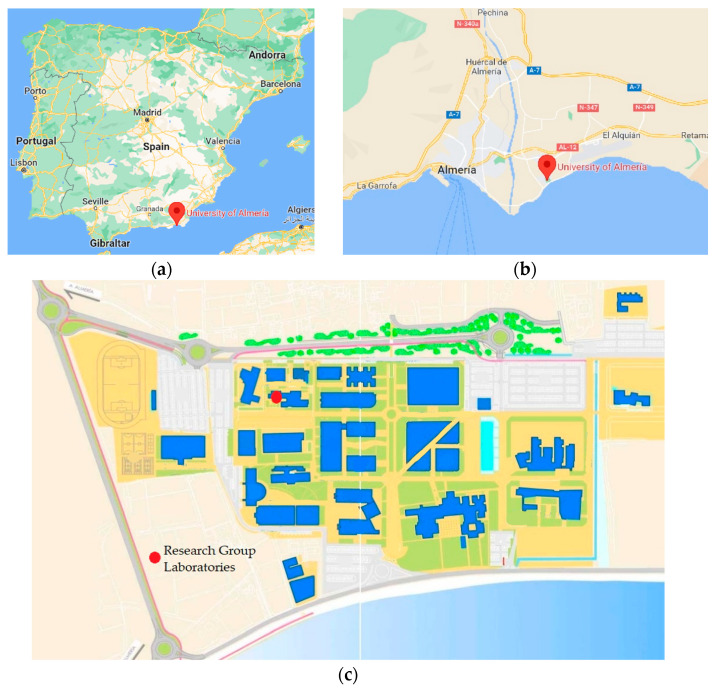
Location of the ELF magnetometer. (**a**) Almeria location; (**b**) UAL location; (**c**) Laboratory location inside the UAL campus.

Data were collected from an ELF magnetometer installed on the campus of the University of Almeria (UAL), Spain, in a laboratory of the Electronics, Communications, and Telemedicine TIC019 Research Group (see Figure 3).

**Figure 4:**
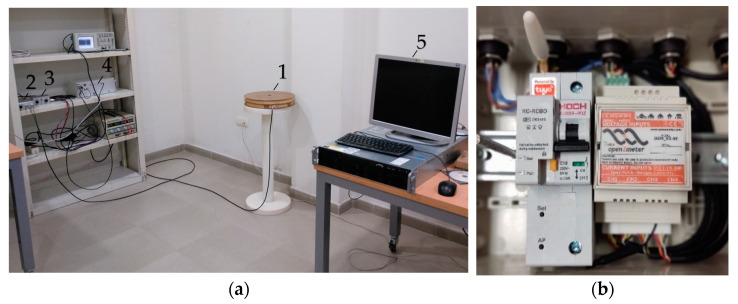
(**a**) System installed in the laboratory of the Research Group: 1. ELF magnetometer; 2. Amplification stage; 3. End amplifier; 4. ADC + DSP; 5. Compute server; (**b**) Front view of the oZm.

The ELF magnetometer allows the measurement of weak EMF of very low frequency and intensity. The geometry, structure, and materials of the system determine its properties (sensitivity, inductance, self-resonance, and bandwidth). The sensor consists of a coreless coil with 12,000 turns of 0.25 mm diameter enameled copper wire. The average diameter of the coil is 32 cm, and the winding width is 4.5 cm. The development of a coil with a large number of turns is not an innovation, although it is of interest due to its size, the wire used, and its configuration [45]. The inductance of the sensor is 68.5 H, and the self-resonance frequency is 990 Hz. The maximum sensitivity of the sensor, plus the amplification stage, is 2.93 mV/pT at 632 Hz, while at 50 Hz the value is 57 µV/pT. Similar sensors were described in previous studies [45]. The analog signal is converted to digital using a 24-bit resolution analog-to-digital converter (ADC). The output of the ADC is read by an embedded digital signal processor (DSP), which performs a pre-processing that consists of decimating the sample rate in the ADC, reducing the acquisition noise [46] and the final system sampling frequency to 4439 Hz. Hence, the bandwidth is half of this frequency, 2219.5 Hz. In the article by Gazquez et al. [47], an electric diagram with the structure of the measurement system can be found. Finally, data are sent via ethernet to a data server. The laboratory also has a compute server, which was in operation between 1 May and 15 May 2021, and was turned off from 16 May onwards to observe the possible effects of the EMF emitted by the server on our magnetometer. The different parts of the system are shown in Figure 4a.

To contrast the values of the grid frequency obtained through the PLHR analysis, the results were compared with data from a commercial power quality analyzer installed in the main electrical switch of the building where the ELF magnetometer was installed. The chosen model was the Openzmeter (oZm), which shows the aggregated mean value of the grid frequency every 3 min. The oZm is a low-cost smart meter with an open-source system (free software and open hardware [48]) developed between the UAL and the University of Granada to help with smart energy metering and power quality analysis in power networks. It measures RMS voltages with an accuracy of up to 0.1%, frequency up to 10 mHz (between 42.5 and 57.5 Hz), and RMS currents according to different sensor probes (the basic version can handle up to 265 V_RMS_ and 35 A_RMS_ using an onboard Hall effect sensor) [49,50]. All features comply with international standards IEC 61000-4-30:2015 [43] and EN 50160 [30] and it has been cited in numerous international research articles [51,52,53,54] and used in research applications [55]. Figure 4b presents a general view of the oZm used in this article.

### 2.2. Frequency Measurement Methods

According to the international standard IEC 61000-4-30:2015 [43], the value of the measurement of the fundamental frequency is the relation between the number of integral cycles counted during 10 s time clock interval *N* divided by the duration of the integer cycles *t* (see Equation (1)). The standard also allows other techniques with equivalent results. In Shilpa et al. [56], different frequency measurement methods by signal processing techniques are described.

(1) $$f=\frac{N_{t=10}}{t}$$

The first method used in this article to obtain the grid frequency through the PLHR analysis was a zero-crossing (ZC) algorithm. A second method was implemented, and an algorithm based on the Fast Fourier transform (FFT) of the signal was used. Even though aggregation is not mandatory according to the standard, these data were aggregated to obtain a value every 3 min to compare with data obtained from the oZm analyzer. The aggregation was calculated using the algorithm mentioned in the standard.

**Figure 5:**
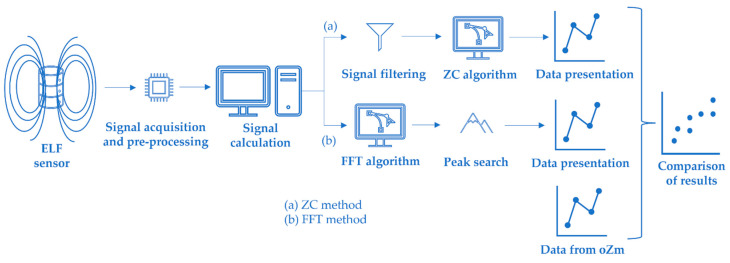
Diagram of the ZC and FFT algorithm used in this paper.

As a final step, the results of the measurements of both methods were compared with data provided by the oZm analyzer (see Figure 5).

#### 2.2.1. Zero-Crossing Method

According to standard IEC 61000-4-30:2015 [43], when this method is used, harmonics and interharmonics must be attenuated to minimize the effects of multiple zero-crossings. The measurement time intervals must not overlap and the individual cycles that overlap the 10 s time clock are discarded. As mentioned in the standard, for some applications such as wind turbines, the use of times shorter than 10 s may eventually be useful and the frequency is obtained from 10/12 cycles frequency (dividing the cycles by the duration of the integer periods).

**Figure 6:**
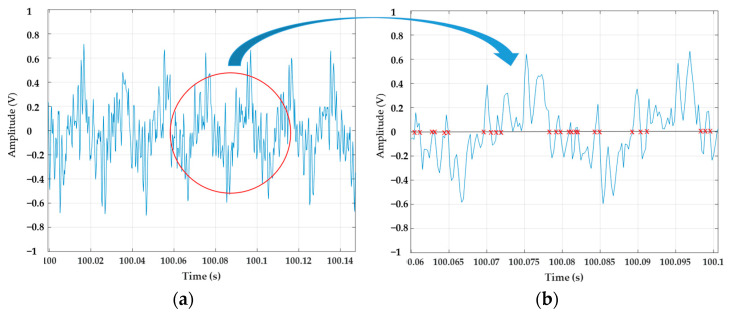
(**a**) Multiple zero−crossings of the time signal; (**b**) Detail.

The features in the time domain are very common due to their computational simplicity. In addition, they are easy to implement since they do not need any transformation [57]. Therefore the ZC method is a practical and efficient way to calculate the frequency of a sampled data series [58]. Previously, a low-pass digital filter was applied to attenuate harmonics and interharmonics to remove undesirable multiple zero-crossings of the time signal obtained in the ELF magnetometer, as can be seen in Figure 6. After the filtering process, the algorithm was applied to find the frequency every 10 s.

#### 2.2.2. FFT Method

**Figure 7:**
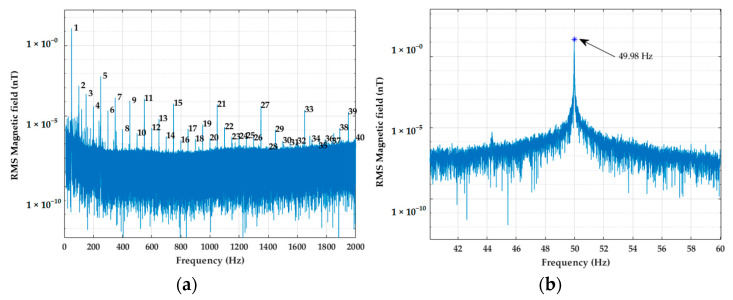
(**a**) Peak search; (**b**) Peak near the fundamental component.

To obtain the Fourier transform, the signal was processed using the FFT. Then, our algorithm searched the frequency of the spectral bar where the maximum values near the fundamental component and its harmonics are reached. Figure 7a shows the numbered peaks corresponding to the fundamental frequency and its harmonics up to the 40th order. Finally, the value of the frequency where the peak is reached was stored (Figure 7b). In order to compare the results with data obtained from the oZm analyzer, a 3-min window was chosen for the FFT method.

## 3. Results and Discussion

**Figure 8:**
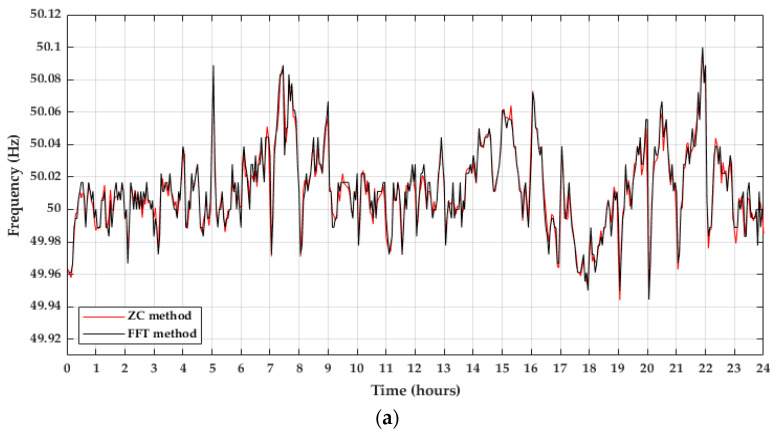

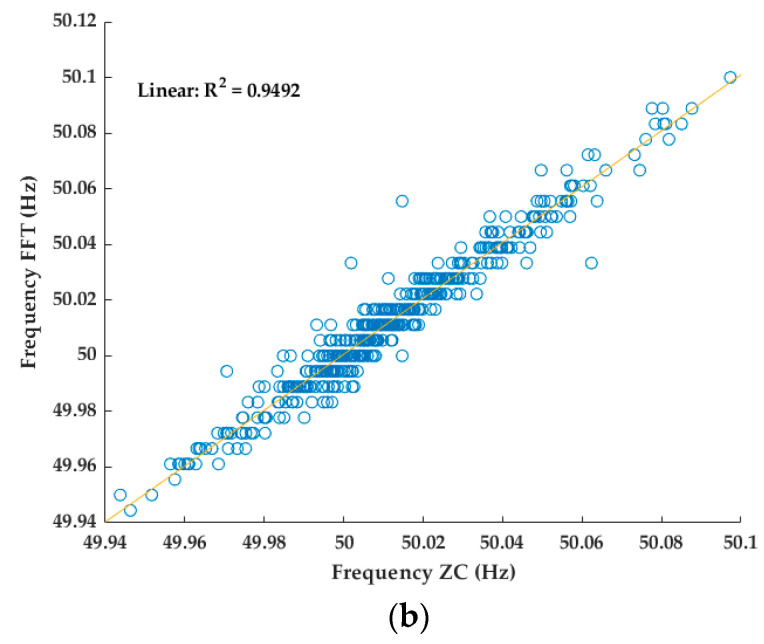
Comparison between ZC and FFT methods. (**a**) Daily frequency of 3 May 2021; (**b**) Linear regression.

All timescales in this paper were expressed in universal coordinated time (UTC), although the local time in Spain in May corresponds to UTC+2. The methods used in this article to obtain the grid frequency through the PLHR analysis were the ZC and the FFT. The results of both methods were compared and represented in Figure 8a for 3 May 2021. Additionally, linear regression in R was carried out for this day and presented in Figure 8b.

The results for both methods were similar, with a coefficient of determination ranging from 0.8600 (day 10th) to 0.9392 (day 30th).

**Figure 9:**
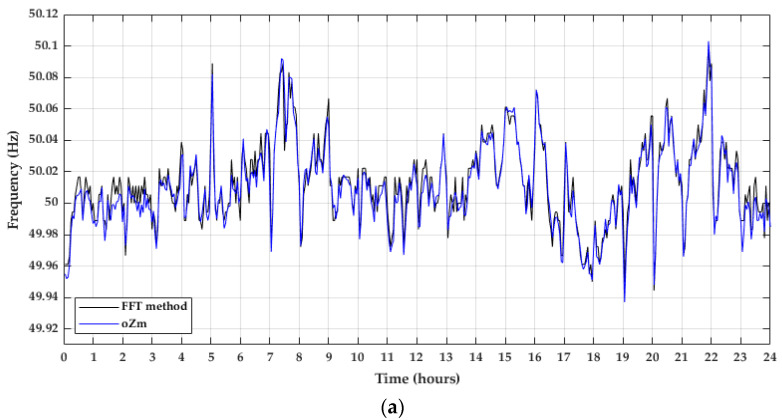

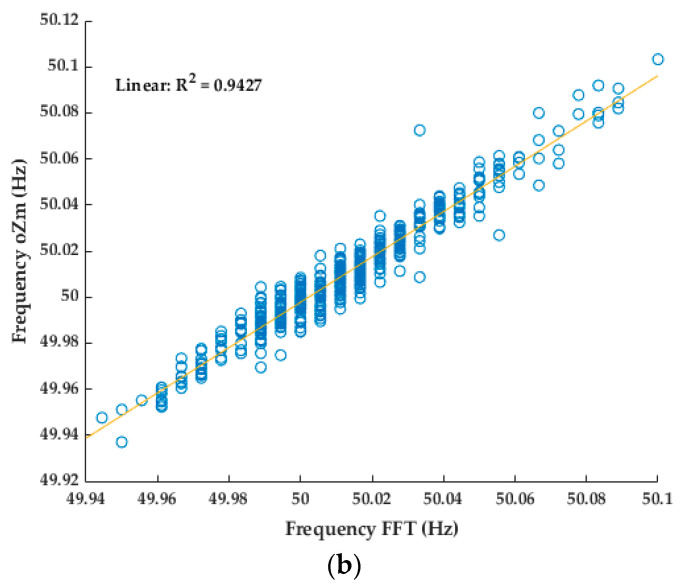
Comparison between the FFT method and the oZm. (**a**) Daily frequency of 3 May 2021; (**b**) Linear regression.

These two methods were applied to the data provided by the ELF sensor to obtain the grid frequency. After that, the results of both methods were compared with the results of the frequency obtained by the commercial analyzer oZm. Firstly, the daily frequency obtained through the FFT method was compared with the frequency registered by the oZm. Data obtained for 3 May 2021 were represented in Figure 9a, as well as the coefficient of determination (Figure 9b).

**Figure 10:**
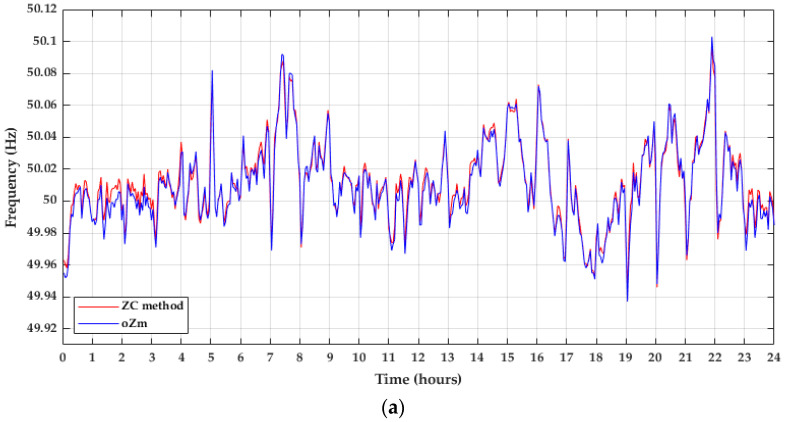

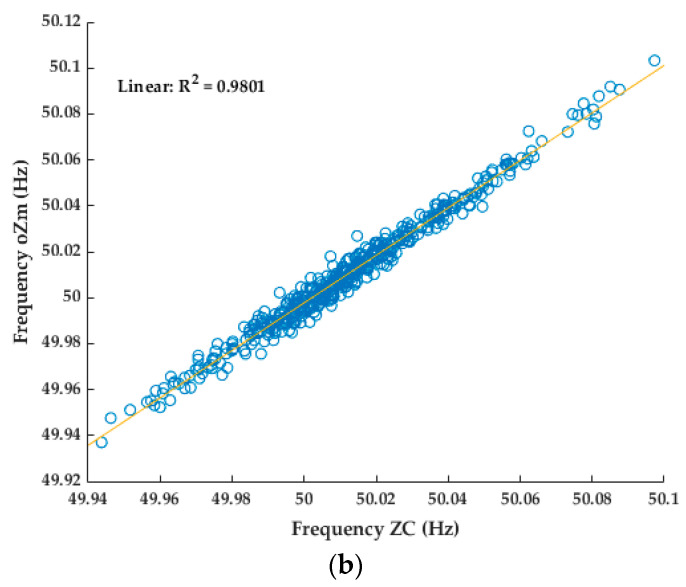
Comparison between the ZC method and the oZm. (**a**) Daily frequency of 3 May 2021; (**b**) Linear regression.

Secondly, the daily frequency obtained through the ZC method was compared with the frequency registered by the oZm. Similarly, data obtained for 3 May 2021 were represented in Figure 10a, as well as the coefficient of determination (Figure 10b).

The coefficient of determination for these comparisons is presented in Table 1 for the complete month of May 2021. According to this, the frequency values obtained from the ZC method fit better with the frequency measured by the oZm analyzer than the results from the FFT method. The coefficient of determination for the comparison between the ZC method and the oZm ranged from 0.6495 (day 25th) to 0.9801 (day 3rd). The coefficient of determination for the comparison between the FFT method and the oZm ranged from 0.5304 (day 25th) to 0.9427 (day 3rd).

**Figure 11:**
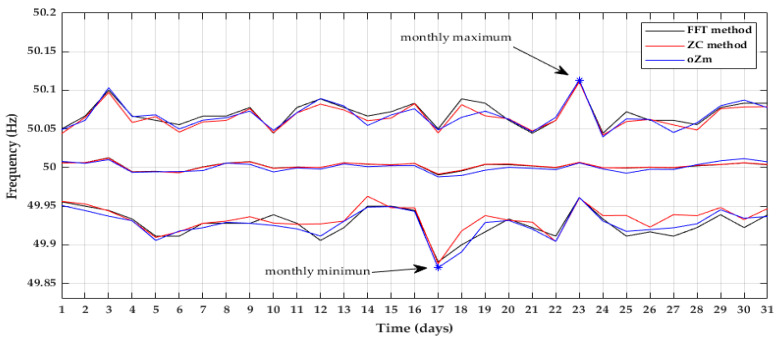
Maximum, minimum, and mean frequency per day. May 2021.

In a monthly analysis, the minimum, mean, and maximum value of the grid frequency per day was presented in Figure 11. The maximum value was reached on the 23rd and the minimum on the 17th.

**Figure 12:**
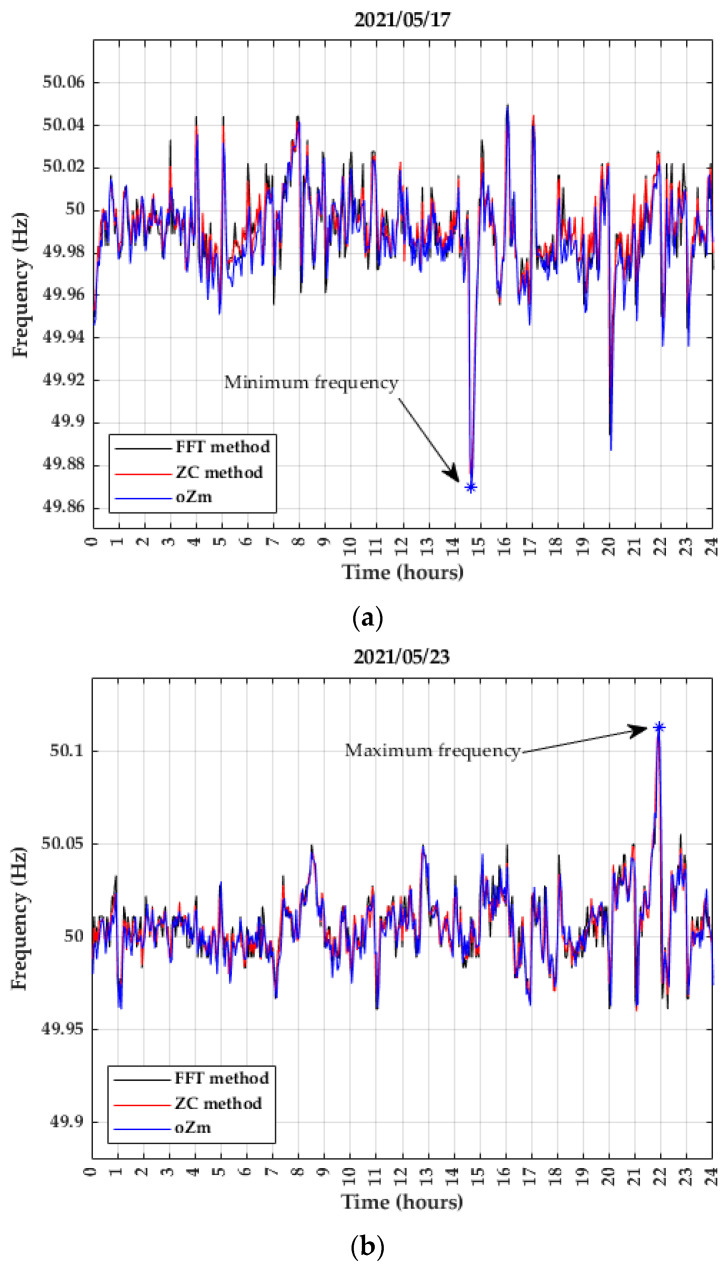
Daily grid frequency variation (**a**) Monthly minimum value of May 2021; (**b**) Monthly maximum value of May 2021.

A detailed analysis for the 17th is shown in Figure 12a. At 14:39, the oZm analyzer registered the minimum value, 49.870 Hz. At the same time, the ELF magnetometer registered 49.876 Hz (ZC method) and 49.878 Hz (FFT method). These values are 0.0120% and 0.0160% higher than the oZm value, respectively.

The maximum value registered by the oZm analyzer is shown in Figure 12b and occurred on day 23rd. The value was 50.113 Hz at 21:54 h. At this time, the sensor registered 50.111 Hz (both methods), a 0.0040% lower value.

The minimum and maximum values of the frequency in May 2021 were within the limits of 50 Hz ± 1% established in the standard. The maximum deviation in the absolute value of the frequency from its nominal value measured by the ELF magnetometer occurred on day 17th, 0.248%, far from the 1% allowed.

PLHR research currently covers a wide variety of fields, the main ones being medicine, astrophysics, and engineering. In the field of medicine, the publications are focused on the possible effects that ELF radiation has on human health. Possible disturbances of the immune system [59] have been collected due to EMF fields. Among the conclusions, this article highlights that it is possible that chronic EMF exposure can lead to chronic allergic, immune dysfunction, and inflammatory responses if it occurs continuously over time. Other studies have considered possible biological effects of ELF radiation, such as changes in the mobility of human sperm [60], modifications in the genetic material [61], effects on the cardiovascular system [62], influence on the functional state of the brain [63], and the possible relationship with sleep disorders [64].

In the field of astrophysics, PLHR are studied in the wave-particle interaction region [65,66]. Nonlinear interactions between PLHR and electrons can induce the precipitation of electrons and part of the PLHR energy dissipates and modifies ionospheric currents [67].

Many other articles monitor the exposure to EMF, in order to verify that regulatory limits are not exceeded and focus their research on how PLHR are becoming a source of EMC problems. In an article from Nicolaou et al. [68], the authors carried out experimental measurements of EMF in open air 132/11 kV substations, obtaining values of 45.89, 38.11, and 35.30 µT (values below the safety guidelines), but in one of the coil rooms, they found values 6.26 times above the safety guidelines.

To our knowledge, there are no previous works relating PLHR and grid frequency. This article focuses on the validation of the results to measure the frequency in a non-invasive way through a PLHR analysis using the available data of the R&D project UAL18-TIC-A025-A corresponding to May 2021.

The procedures implemented in this article used a ZC and an FFT algorithm to obtain the grid frequency every 3 min. The results of both methods were compared between them, providing the best coefficient of determination for 3 May (0.9492). Then, the values of both methods were compared with the frequency obtained from the commercial analyzer oZm. In our study period, we did not see clear advantages in the use of the FFT method with respect to the ZC method to find the grid frequency, since the coefficient of determination was lower in the FFT method than in the ZC method when the results were compared with the oZm. In addition, the computational cost of both methods was similar, since digital filtering must be added in the ZC method, which makes the computational cost similar compared to the FFT method. Considering now the ZC method, 3 May 2021 was the day when the frequency measured by the ELF sensor fit better with the frequency registered by the oZm, with a coefficient of determination of 0.9801.

Due to the presence of the compute server in the laboratory where the sensor was installed, it was decided to turn it off from 16 May 2021 onwards, and no differences were found in the results of both fortnights in the monthly analysis performed. According to this analysis, the maximum deviation in the mean daily frequency occurred on 19 May 2021, with a value of 0.0155%. Similarly, the maximum deviation in the maximum and minimum daily frequency occurred on 18 May 2021, with values of 0.0327% and 0.0543%, respectively.

## 4. Conclusions

Since September 2020, an ELF magnetometer has been monitoring, in real time, the EMF produced by electrical installations, collecting a wealth of information. These data, properly processed, have allowed us to establish a procedure to measure the grid frequency through a PLHR analysis.

After analyzing the results of a full month, specifically, May 2021, the indirect and non-invasive measurement of the grid frequency through the PLHR analysis successfully fits the grid frequency with the ZC and the FFT methods. The validation procedure with the commercial device produced a coefficient of determination for the ZC method ranging from 0.9801 on 3 May 2021, to 0.6495 on 25 May 2021. For the FFT method, it ranged from 0.9427 on 3 May 2021, to 0.5304 on 25 May 2021.

Nowadays, the electrical power system is one of the largest and most widely established man-made objects, with billions of components. It has become more and more complex, and modern energy management systems have a strong focus on power stability monitoring. Frequency is key in power systems since deviations from the nominal value may affect power system operations and damage equipment. Although the maximum deviation found in May 2021 of the frequency from its nominal value was 0.248%, far from the limits established in the standard, a recent problem is the fluctuations that the massive implementation of wind or photovoltaic generation introduces in frequency.

Accordingly, accurate measurements are required as electrical demand continues to increase. Frequency measurement is usually carried out using direct methods that require a connection to the distribution network. In our case study, PLHR are obtained through the EMF generated by electrical installations; thus, we can measure the grid frequency without a point of connection, performing the measurement non-invasively. The advantage is that it is possible to monitor the grid frequency of a wide area, with portable equipment that does not require a physical connection, and without the need to disturb the users of the electrical power network.

For future works, the system could be optimized for grid frequency measurement if an analog lowpass filter is implemented to attenuate harmonics and interharmonics before the signal processing. Thus, the ZC method could be carried out with a lower computational cost because of the absence of the digital filtering stage, which would be another advantage over the FFT method. Another improvement would be to make the entire system compact and portable. This would require reducing the size of the sensor, which could be achieved by using ultra-thin wire and a high permeability ferrite core for the coil. With these possible improvements, progress would be made in monitoring the power network quality and would contribute toward a better understanding of the behavior of the PLHR.

## Figures and Tables

**Table 1:** Coefficient of determination R^2^ of ZC vs. oZm, and FFT vs. oZm of May 2021.

Day	1	2	3	4	5	6	7	8	9	10	11	12	13	14	15	16
ZC vs. oZm	0.9587	0.9185	0.9801	0.9560	0.9626	0.9377	0.9532	0.9436	0.9414	0.9855	0.9311	0.9145	0.7207	0.8630	0.8920	0.8846
FFT vs. oZm	0.8787	0.8280	0.9427	0.8963	0.8992	0.8651	0.8735	0.8689	0.8799	0.8110	0.8409	0.8083	0.6521	0.6817	0.7748	0.7686
	17	18	19	20	21	22	23	24	25	26	27	28	29	30	31	
ZC vs. oZm	0.8763	0.8785	0.8323	0.8698	0.8208	0.8048	0.8702	0.8310	0.6495	0.7177	0.7109	0.7367	0.7577	0.8089	0.7713	
FFT vs. oZm	0.7767	0.7602	0.6714	0.7432	0.6833	0.6694	0.7663	0.7180	0.5304	0.6413	0.5717	0.6307	0.6499	0.7085	0.6454	

## References

[1] Kufeoglu S., Pollitt M., Anaya K. (2018). Electric Power Distribution in the World: Today and Tomorrow. EPRG-Camb. Work. Pap. Econ..

[2] Wu J., Guo Q., Yue C., Xie L., Zhang C. (2020). Special Electromagnetic Interference in the Ionosphere Directly Correlated with Power System. IEEE Trans. Electromagn. Compat..

[3] Němec F., Parrot M., Santolík O. (2015). Power line harmonic radiation observed by the DEMETER spacecraft at 50/60 Hz and low harmonics. J. Geophys. Res. A Space Phys..

[4] Helliwell R.A., Katsufrakis J.P. (1974). VLF wave injection into the magnetosphere from Siple Station, Antarctica. J. Geophys. Res..

[5] Nunn D., Rycroft M., Trakhtengerts V. (2005). A parametric study of the numerical simulations of triggered VLF emissions. Ann. Geophys..

[6] Helliwell R.A., Katsufrakis J.P., Bell T.F., Raghuram R. (1975). VLF line radiation in the Earth’s magnetosphere and its association with power system radiation. J. Geophys. Res..

[7] Burke C.P., Jones D.L. (1992). An experimental investigation of ELF attenuation rates in the Earth-ionosphere duct. J. Atmos. Terr. Phys..

[8] Wang Y., Zhou Y., Cao Q. (2014). Study of ELF propagation parameters based on the simulated schumann resonances. IEEE Antennas Wirel. Propag. Lett..

[9] National Centers for Environmental Information Geomagnetic Coordinates. https://www.ngdc.noaa.gov/geomag/WMM/icons/WMM2010_coor.png.

[10] García R.M., Novas N., Alcayde A., El Khaled D., Fernández-Ros M., Gazquez J.A. (2020). Progress in the knowledge, application and influence of extremely low frequency signals. Appl. Sci..

[11] Xi G., Yang Y.J., Liu K., Zhang X.H. (2012). Effect of extremely low frequency pulsed electric field based on plant potential fluctuations on growth of mung bean. Gaodianya Jishu/High Volt. Eng..

[12] Wu J., Guo Q., Yan X., Zhang C. (2019). Theoretical analysis on affecting factors of power line harmonic radiation. IEEE Trans. Plasma Sci..

[13] Red Eléctrica de España (2020). El Sistema Eléctrico Español. https://www.ree.es/sites/default/files/publication/2021/06/downloadable/inf_sis_elec_ree_2020_0.pdf.

[14] Zhang C., Ma Q. (2018). Influences of radiation from terrestrial power sources on the ionosphere above China based on satellite observation. IOP Conf. Ser. Earth Environ. Sci..

[15] Amirkhanyan M., Bryukhan F. (2018). Measurement errors of electromagnetic fields of industrial frequency in urban areas. MATEC Web Conf..

[16] Belyaev I., Dean A., Eger H., Hubmann G., Jandrisovits R., Kern M., Kundi M., Moshammer H., Lercher P., Müller K. (2016). EUROPAEM EMF Guideline 2016 for the prevention, diagnosis and treatment of EMF-related health problems and illnesses. Rev. Environ. Health.

[17] Ahlbom A., Cardis E., Green A., Linet M., Savitz D., Swerdlow A. (2001). Review of the epidemiologic literature on EMF and health. Environ. Health Perspect..

[18] Persinger M.A., Forster W.S. (1970). ELF rotating magnetic fields: Prenatal exposure and adult behavior. Arch. Meteorol. Geophys. Bioklimatol. Ser. B.

[19] Wertheimer N., Leeper E. (1979). Electrical wiring configurations and childhood cancer. Am. J. Epidemiol..

[20] Barker A. (2002). The possible biological effects of low-frequency electromagnetic fields. Supplement to the Public Affairs Board Report No. 10 June 1994. IEEE Electr. Insul. Mag..

[21] Lacy-hulbert A., Metcalfe J.C., Hesketh R. (1998). Biological responses to electromagnetic fields. FASEB J..

[22] WHO (2007). Extremely Low Frequency Fields, Environmental Health Criteria Monograph No. 238.

[23] Rachedi B.A., Babouri A., Xun Z. (2016). Electromagnetic pollution inside high voltage substation. Rev. Roum. Sci. Techn. Electrotech. et Énerg..

[24] Tardón A., Velarde H., Rodriguez P., Moreno S., Raton M., Muñoz J., Fidalgo A.R., Kogevinas M. (2002). Exposure to extremely low frequency magnetic fields among primary school children in Spain. J. Epidemiol. Community Health.

[25] Aris A., Yiannis K., Tyrakis C., Alkhorayef M., Sulieman A., Tsougos I., Theodorou K., Kappas C. (2020). Extremely low frequency electromagnetic field exposure measurement in the vicinity of wind turbines. Radiat. Prot. Dosim..

[26] Werner S.M., Rodger C.J., Thomson N.R. (2005). Identifying power line harmonic radiation from an electrical network. Ann. Geophys..

[27] Wu J., Fu J.J., Zhang C. (2014). Propagation characteristics of power line harmonic radiation in the ionosphere. Chin. Phys. B.

[28] Němec F., Santolík O., Parrot M., Berthelier J.J. (2007). Power line harmonic radiation: A systematic study using DEMETER spacecraft. Adv. Space Res..

[29] Haehne H., Schottler J., Waechter M., Peinke J., Kamps O. (2018). The footprint of atmospheric turbulence in power grid frequency measurements. EPL.

[30] European Committee for Electrotechnical Standarization (CENELEC) (2010). EN 50160:2010—Voltage Characteristics of Electricity Supplied by Public Distribution Networks.

[31] European Committee for Electrotechnical Standarization (CENELEC) (2015). EN 50160:2010/A1:2015—Voltage Characteristics of Electricity Supplied by Public Distribution Networks.

[32] European Committee for Electrotechnical Standarization (CENELEC) (2019). EN 50160:2010/A2:2019—Voltage Characteristics of Electricity Supplied by Public Distribution Networks.

[33] European Committee for Electrotechnical Standarization (CENELEC) (2019). EN 50160:2010/A3:2019—Voltage Characteristics of Electricity Supplied by Public Distribution Networks.

[34] Alam K., Chakraborty T., Pramanik (Chaudhury) S., Sarddar D., Mal S. (2013). Measurement of Power Frequency with Higher Accuracy Using PIC Microcontroller. Procedia Technol..

[35] Grigsby L.L. (2017). Power System Stability and Control.

[36] Rydin Gorjão L., Jumar R., Maass H., Hagenmeyer V., Yalcin G.C., Kruse J., Timme M., Beck C., Witthaut D., Schäfer B. (2020). Open database analysis of scaling and spatio-temporal properties of power grid frequencies. Nat. Commun..

[37] Hartmann B., Vokony I., Táczi I. (2019). Effects of decreasing synchronous inertia on power system dynamics—Overview of recent experiences and marketisation of services. Int. Trans. Electr. Energy Syst..

[38] Wu Z., Gao W., Gao T., Yan W., Zhang H., Yan S., Wang X. (2018). State-of-the-art review on frequency response of wind power plants in power systems. J. Mod. Power Syst. Clean Energy.

[39] Ashraf S.M., Gupta A., Choudhary D.K., Chakrabarti S. (2017). Voltage stability monitoring of power systems using reduced network and artificial neural network. Int. J. Electr. Power Energy Syst..

[40] Kumar C.S., Ramesh P., Kasilingam G., Ragul D., Bharatiraja C. (2021). The power quality measurements and real time monitoring in distribution feeders. Mater. Today Proc..

[41] Abdulraheem B.S., Gan C.K. (2016). Power system frequency stability and control: Survey. Int. J. Appl. Eng. Res..

[42] Bevrani H., Raisch J. (2017). On Virtual inertia Application in Power Grid Frequency Control. Energy Procedia.

[43] International Electrotechnical Commission (IEC) (2015). IEC 61000-4-30:2015—Electromagnetic Compatibility (EMC)—Part 4–30: Testing and Measurement Techniques—Power Quality Measurement Methods.

[44] International Electrotechnical Commission (IEC) (2016). IEC 61000-4-30:2015/COR1:2016—Electromagnetic Compatibility (EMC)—Part 4–30: Testing and Measurement Techniques—Power Quality Measurement Methods.

[45] García R.M., Gázquez J.A., Novas N. (2013). Characterization and modeling of high-value inductors in ELF band using a vector network analyzer. IEEE Trans. Instrum. Meas..

[46] Fernández-Ros M., Gázquez J.A., García R.M., Novas N. (2016). Optimization of the periodogram average for the estimation of the power spectral density (PSD) of weak signals in the ELF band. Meas. J. Int. Meas. Confed..

[47] Gázquez J.A., Fernández-Ros M., Novas N., García R.M. (2015). Techniques for Schumann Resonance Measurements: A Comparison of Four Amplifiers with a Noise Floor Estimate. IEEE Trans. Instrum. Meas..

[48] openZmeter What Is openZmeter? Smart Metering and Power Quality Analysis for the People. https://openzmeter.com/.

[49] Viciana E., Alcayde A., Montoya F.G., Baños R., Arrabal-Campos F.M., Zapata-Sierra A., Manzano-Agugliaro F. (2018). OpenZmeter: An efficient low-cost energy smart meter and power quality analyzer. Sustainability.

[50] Viciana E., Alcayde A., Montoya F.G., Baños R., Arrabal-Campos F.M., Manzano-Agugliaro F. (2019). An open hardware design for internet of things power quality and energy saving solutions. Sensors.

[51] Chen Y.Y., Lin Y.H. (2019). A smart autonomous time-and frequency-domain analysis current sensor-based power meter prototype developed over fog-cloud analytics for demand-side management. Sensors.

[52] Chen Y.Y., Lin Y.H., Kung C.C., Chung M.H., Yen I.H. (2019). Design and implementation of cloud analytics-assisted smart power meters considering advanced artificial intelligence as edge analytics in demand-side management for smart homes. Sensors.

[53] Luque J., Anguita D., Pérez F., Denda R. (2020). Spectral analysis of electricity demand using hilbert–huang transform. Sensors.

[54] Artale G., Caravello G., Cataliotti A., Cosentino V., Di Cara D., Dipaola N., Guaiana S., Panzavecchia N., Sambataro M.G., Tinè G. (2020). Pq and harmonic assessment issues on low-cost smart metering platforms: A case study. Sensors.

[55] Montoya F.G., Baños R., Alcayde A., Arrabal-Campos F. Efficient open-source power quality analyser and smart meter. Proceedings of the 25th International Conference on Electricity Distribution CIRED 2019.

[56] Sondkar S.Y., Dudhane S., Abhyankar H.K. (2012). Frequency measurement methods by signal processing techniques. Procedia Eng..

[57] Waris A., Niazi I.K., Jamil M., Gilani O., Englehart K., Jensen W., Shafique M., Kamavuako E.N. (2018). The effect of time on EMG classification of hand motions in able-bodied and transradial amputees. J. Electromyogr. Kinesiol..

[58] Toledo-Perez D.C., Rodriguez-Resendiz J., Gomez-Loenzo R.A. (2020). A study of computing zero crossing methods and an improved proposal for EMG signals. IEEE Access.

[59] Johansson O. (2009). Disturbance of the immune system by electromagnetic fields-A potentially underlying cause for cellular damage and tissue repair reduction which could lead to disease and impairment. Pathophysiology.

[60] Górski R., Kotwicka M., Skibińska I., Jendraszak M., Wosiński S. (2020). Effect of low-frequency electric field screening on motilityof human sperm. Ann. Agric. Environ. Med..

[61] Wolf F.I., Torsello A., Tedesco B., Fasanella S., Boninsegna A., D’Ascenzo M., Grassi C., Azzena G.B., Cittadini A. (2005). 50-Hz extremely low frequency electromagnetic fields enhance cell proliferation and DNA damage: Possible involvement of a redox mechanism. Biochim. Biophys. Acta-Mol. Cell Res..

[62] McNamee D.A., Legros A.G., Wisenberg G., Prato F.S., Thomas A.W., Krewski D.R. (2009). A literature review: The cardiovascular effects of exposure to extremely low frequency electromagnetic fields. Int. Arch. Occup. Environ. Health.

[63] Lyskov E.B., Juutilainen J., Jousmäki V., Partanen J., Medvedev S., Hänninen O. (1993). Effects of 45-Hz magnetic fields on the functional state of the human brain. Bioelectromagnetics.

[64] Warille A.A., Onger M.E., Turkmen A.P., Deniz G., Altun G., Yurt K.K., Altunkaynak B.Z., Kaplan S. (2016). Controversies on electromagnetic field exposure and the nervous systems of children. Histol. Histopathol..

[65] Wu J., Zhang C., Zeng L., Ma Q. (2017). Systematic investigation of power line harmonic radiation in near-Earth space above China based on observed satellite data. J. Geophys. Res. Space Phys..

[66] Parrot M. (2018). DEMETER observations of manmade waves that propagate in the ionosphere. C. R. Phys..

[67] De Santis A., De Franceschi G., Spogli L., Perrone L., Alfonsi L., Qamili E., Cianchini G., Di Giovambattista R., Salvi S., Filippi E. (2015). Geospace perturbations induced by the Earth: The state of the art and future trends. Phys. Chem. Earth.

[68] Nicolaou C.P., Papadakis A.P., Razis P.A., Kyriacou G.A., Sahalos J.N. (2012). Experimental measurement, analysis and prediction of electric and magnetic fields in open type air substations. Electr. Power Syst. Res..

